# Surgical management of complex perianal fistula revisited in a systematic review: a critical view of available scientific evidence

**DOI:** 10.1186/s12893-023-01912-z

**Published:** 2023-02-05

**Authors:** D. García-Olmo, M. Gómez-Barrera, F. de la Portilla

**Affiliations:** 1 New Therapies Laboratory, Health Research Institute-Fundación Jiménez Díaz University Hospital (IIS-FJD/UAM), Department of Surgery, Fundación Jiménez Díaz University Hospital (UAM), Avda. Reyes Católicos, 2, 28040 Madrid, Spain; 2 Pharmacoeconomics & Outcomes Research Iberia (PORIB), Paseo Joaquín Rodrigo, 4 i, 28224 Pozuelo de Alarcón, Madrid Spain; 3 Coloproctology Unit, Clinical Management Unit of General and Gastrointestinal Surgery, Division Seville, Biomedical Research Institute (IBIS), University Hospital Virgen del Rocio/CSIC University of Seville, Seville, Spain

**Keywords:** Crohn’s disease, Complex perianal fistula, Cryptoglandular fistula, Surgery

## Abstract

**Background:**

Treating complex perianal fistulas in Crohn’s disease patients remains a challenge. Classical surgical treatments for Crohn’s disease fistulas have been extrapolated from cryptoglandular fistulas treatment, which have different etiology, and this might interfere with its effectiveness, in addition, they increase fecal incontinence risk. Recently, new surgical techniques with support from biological approaches, like stem cells, have been developed to preserve the function of the sphincter. We have performed a systematic literature review to compare the results of these different techniques in the treatment of Crohn’s or Cryptoglandular fistula.

**Methods:**

PubMed, EMBASE, Database of Abstracts of Reviews of Effectiveness, Cochrane Central Register of Controlled Trials were searched systematically for relevant articles. We included randomized controlled trials and observational studies that referred to humans, were written in English, included adults 18+ years old, and were published during the 10-year period from 2/01/2010 to 2/29/2020. Evidence level was assigned as designated by the Scottish Intercollegiate Guidelines Network.

**Results:**

Of the 577 citations screened, a total of 79 were ultimately included in our review. In Crohn’s disease patients, classical techniques such as primarily seton, Ligation of Intersphincteric Fistula Tracks, or lay open, healing rates were approximately 50–60%, while in cryptoglandular fistula were around, 70–80% for setons or flaps. In Crohn’s disease patients, new surgical techniques using derivatives of adipose tissue reported healing rates exceeding 70%, stem cells-treated patients achieved higher combined remission versus controls (56.3% vs 38.6%, p = 0.010), mesenchymal cells reported a healing rate of 80% at week 12. In patients with cryptoglandular fistulas, a healing rate of 70% using derivatives of adipose tissue or platelets was achieved, and a healing rate of 80% was achieved using laser technology. Fecal incontinence was improved after the use of autologous platelet growth factors and Nitinol Clips.

**Conclusion:**

New surgical techniques showed better healing rates in Crohn’s disease patients than classical techniques, which have better results in cryptoglandular fistula than in Crohn’s disease. Healing rates for complex cryptoglandular fistulas were similar between the classic and new techniques, being the new techniques less invasive; the incontinence rate improved with the current techniques.

**Supplementary Information:**

The online version contains supplementary material available at 10.1186/s12893-023-01912-z.

## Background

The estimated incidence of perianal fistulas in Europe is 1.2–2.8 per 10,000 people [1–3]. Perianal fistulas appear in 30–50% of Crohn’s disease (CD) cases, and 80% of those fistulas are classified as complex [4, 5]. In this scenario, medical treatments are intended to promote long-term fistula healing, while preserving continence and avoiding diverting stomas [6]. However, these goals are often unmet with currently available therapies, particularly in relation to complex perianal fistulas, which are the most challenging to treat [7]. Treatment of complex perianal fistulas (CPF) in patients with CD is especially challenging for surgeons and gastroenterologists. Medical therapy is typically still recommended as a first line treatment, with surgery being reserved for sepsis control or laying open superficial tracks [8, 9].

During the last 30 years, many “classic” surgical techniques used to treat cryptoglandular complex perianal fistulas, such as core out, advancement flaps, or ligation of intersphincteric fistula tracks (LIFTs), have also been used to resolve CPF associated with CD [10]. However, during the last 10 years, a shift has occurred to a new and usually minimally invasive surgery (MIS), with support from biological approaches, such as stem cells, platelet rich plasma, or the use of fibrin or glue, to avoid touching the sphincter, hence preserving fecal continence.

The aim of this study was to conduct a systematic review and audit of the results of complex perianal fistula surgeries for patients with CD or cryptoglandular fistulas. We performed this review to revisit the concept of perianal complex fistula treatments and answer questions concerning the clinical evidence that has emerged regarding various surgical approaches to CD and cryptoglandular complex perianal fistulas, in terms of fistula healing, Health-Related Quality of Life (HRQoL), cost, and fecal continence.

## Methods

### Review design

The protocol (stored in PORIB) and reporting methodology for this systematic review was designed in accordance with the PRISMA-P guidelines [11]. The participants, interventions, comparisons, outcomes, and study design (PICO) strategy was followed to identify the populations (CPF in patients with CD or fistulas of cryptoglandular origin), intervention (surgery), comparisons (clinical trials or observational studies), and outcomes (clinical, economic, and quality of life) (Additional file 1).

### Eligibility criteria

We included all the primary studies published in the medical literature related to the clinical outcomes, quality of life, or economic costs of complex perianal fistula surgeries for patients with CD or cryptoglandular-associated fistulas not related to CD. The eligible studies included randomized controlled trials (RCTs) and observational studies that referred to humans, were written in English, included adults 18+ years old, and were published in the 10-year period from 2/01/2010 to 2/29/2020. We excluded articles regarding a different population (Reason 1), type of fistula such as simple fistulas, internal fistulas, such as rectovaginal, anovaginal, rectourethral, or ileovaginal fistulas (Reason 2), non-surgical interventions, such as pharmacological or medical treatments (Reason 3), type of study such as reviews (Reason 4) and publications without clinical outcome, quality of life, or cost results (Reason 5).

### Data sources and search strategy

In March 2020, a literature search strategy was designed using variations on the search terms “Crohn’s disease”, “Rectal fistula”, “Perianal”, “Fistulizing disease”, “Complex”, “Inflammatory Bowel Disease”, “Cryptoglandular”, “Surgical intervention”, and “Surgical procedures”. The following databases were searched: PubMed, EMBASE, the Database of Abstracts of Reviews of Effectiveness (DARE), and the Cochrane Central Register of Controlled Trials (CENTRAL). We also searched the 2018–2020 abstract books for the European Society of Coloproctology (ESCP), European Crohn’s and Colitis Organisation (ECCO), United European Gastroenterology (UEG) Week, American Gastroenterological Association (AGA), and the American Society of Colon & Rectal Surgeons. The search strategy is shown in Additional file 2.

### Study selection and data extraction

Two reviewers independently reviewed each citation against our eligibility criteria in the following 2-stage process: (1) title and abstract and (2) full text. During the study selection and data extraction stages, disagreements between the reviewers were resolved through discussion or with a decision made by a third researcher.

### Quality assessment

Each article was assigned one of the following quality of study scores, as designated by the Scottish Intercollegiate Guidelines Network (SIGN) [12], according to the level of evidence provided in the paper:

- 1++: High quality meta-analyses, systematic reviews of RCTs, or RCTs with an extremely low risk of bias
- 1+: Well-conducted meta-analyses, systematic reviews, or RCTs with a low risk of bias Meta-analyses, systematic reviews, or RCTs with a high risk of bias
- 2++: High quality systematic reviews of case control or cohort studies.
- High quality case control or cohort studies with a very low risk of confounding factors or bias and a high probability that the relationship is causal.
- 2+: Well-conducted case control or cohort studies with a low risk of confounding factors or bias and a moderate probability that the relationship is causal.
- Case control or cohort studies with a high risk of confounding factors or bias and a significant risk that the relationship is not causal.
- 3: Non-analytic studies, e.g. case reports, case series.
- 4: Expert opinions.

We extracted the intervention, study design, number of patients, and main conclusions from each selected article and added the 2019 impact factor from the Journal Citation Reports (JCR) to provide more information for the qualitative analysis.

## Results

**Fig. 1:**
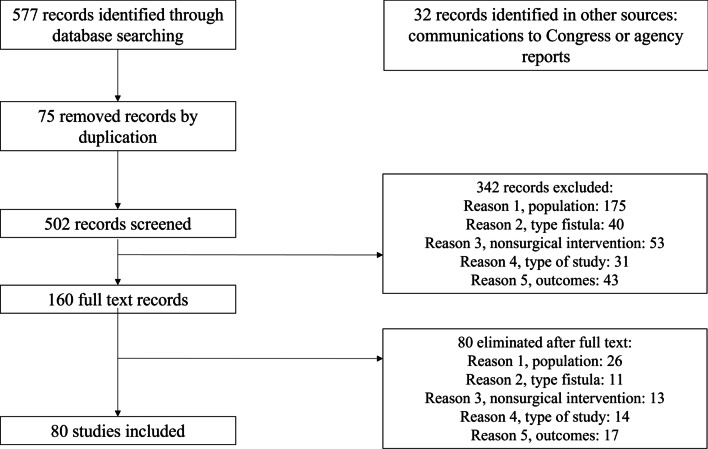
Flow chart of the article selection process

We identified 577 citations from PubMed, EMBASE, DARE, and CENTRAL, and 32 records from other sources (Fig. 1). After removing duplicate papers (75 papers), the titles and abstracts of 502 citations were screened, from which 342 were excluded. Subsequently, the full text of 160 citations was retrieved and assessed for inclusion. Eighty studies were finally retained. No additional studies were identified through tracking citations or by manually reviewing the references of included studies.

We split the results into two categories, i.e., CD-associated CPF and CPF of cryptoglandular origin. We also differentiated between MIS and classic surgical techniques. The clinical results are shown in Tables 1 and 2.

**Table 1:** Results of the Crohn’s disease analysis

Author and year	Design (as appears in the paper)	N	Intervention	Evidence level and IF	Main outcomes
Minimal invasive surgery					
Serrero 2019 [13]	One harm clinical trial	10	Local microinjection combining autologous microfat and adipose-derived stromal vascular fraction	3; 17.373	• Three adverse events were reported of which two required hospitalization • 70% percent of patients had a clinical response at week 12 and 80% at week 48 • 20% and 60% of patients had combined remission at weeks 12 and 48, respectively • Both results confirmed by magnetic resonance imaging • A significant improvement in perianal disease severity was observed with a decreased PDAI score (7.3 at baseline, 3.8 at week 12 and 3.4 at week 48; p = 0.002) and an increased quality of life score (p = 0.038)
Panes 2018 [14]	Randomized, placebo-controlled Phase III clinical trial	212	Stem cell therapy	1++; 17.373	• Patients were randomized to receive administration of adipose stem cells (Cx601) (n = 107) or placebo (n = 105) • At week 52, a significantly greater proportion of patients given Cx601 achieved combined remission (56.3%) versus controls (38.6%) (a difference of 17.7%; 95% CI 4.2–31.2%; p = 0.010), and clinical remission (59.2% versus 41.6% of controls, for a difference of 17.6%; 95% CI 4.1–31.1%; p = 0.013) • Results confirmed by magnetic resonance imaging • Safety was maintained until week 52; adverse events occurred in 76.7% of patients in the Cx601 group and 72.5% patients in the control group
Serrero 2017 [15]	Open, non-comparative, phase I-II, monocentric study	9	Autologous adipose-derived stromal vascular fraction	3; 8.658	• No serious adverse events have been reported • Fistula closure was also evaluated via radiological assessment with magnetic resonance imaging • The only side effect was moderate pain at the lipoid suction site • Efficacy data at week 12 for the first 7 treated patients were 71% response and 28% complete healing, significant reduction in discharge (p < 0.001), significant reduction in the severity of perianal disease (p = 0.045) and significant improvement in quality of life (p = 0.039)
Wilhem 2017 [16]	Prospective study	117	Laser FiLaC®	3; 2.721	• The study analysed 117 patients in a follow-up period of 25.4 months (median) (range 6–60 months) with 13 patients (11.1%) CD-related fistulas • Primary healing rate was 75/117 (64.1%) overall, and 63.5% in cryptoglandular versus 69.2% in CD-fistulas • The secondary healing rate at the end of the study was 103/117 (88.0%) overall and 85.5% for cryptoglandular versus 92.3% associated with CD • There were no differences between the two populations
Dietz 2017 [17]	Phase I clinical trial	12	Autologous mesenchymal stem cells	3; 17.373	• At 6 months, 10/12 patients (83%) presented complete clinical healing and radiographic markers of response • No serious adverse events were related to mesenchymal stem cells
Hermann 2016 [18]	Retrospective observational study	47	Noncutting setons (group 2, n = 23) vs. vacuum-assisted closure (group 1, n = 24) (VAC)	2+; 3.111	• Significant differences were observed between groups (p = 0.006) in the closure of fistulas after 6 months of treatment; 18 patients (75%) group 1 versus 8 patients (35%) Group 2 • Partial response occurred in five patients (21%) from group 1 and in five patients (22%) from group 2 • Closure of fistulous tracts was visualized via magnetic resonance in nine patients (37.50%) from group 1, more than twice the number of patients from group 2 • Results confirmed by magnetic resonance imaging • PDAI scores decreased significantly in both patient groups, from 9 to 1 in patients treated with VAC and from 11 to 5 (p < 0.0001) in patients with seton placement (p < 0.0001) • Preliminary results showed a high closing rate of CD-related fistulas after application of VAC
Panes 2016 [19]	Clinical trial phase III, randomized, double-blind, placebo-controlled	212	Single local administration of stem cells derived from allogenic adipose tissue	1++; 60.392	• 212 patients were randomized: 107 to Cx601 and 105 to placebo • A significantly higher proportion of patients treated with Cx601 achieved combined remission in intention to treat (53 out of 107 [50%] versus 36 out of 105 [34%]; difference 15.2%, CI 97.5% (0.2–30.3; p = 0.024) and in modified intention to treat population (53 out of 103 [51%] vs 36 of 101 [36%]; 15.8%, 0.5–31.2; p = 0.021) • 17% of the patients receiving Cx601 versus 30 (29%) of the 130 in the placebo group experienced treatment-related AE • Results confirmed by magnetic resonance imaging • The most common AE was anal abscess (6 in the Cx601 vs 9 group in the placebo group) and proctalgia (5 vs 9)
Molendijk 2015 [20]	Double-blind placebo-controlled clinical trial	21	Bone marrow mesenchymal mother cells (MMC)	1+; 17.373	• AE were not associated with any of the MMC doses • The random assignment of the treatment was: Group 1 (n = 5) injection of 1 × 10^7^; Group 2 (n = 5) injection of 3 × 10^7^; Group 3 (n = 5) injection of 9 × 10^7^ and placebo (cell less solution) (n = 6) • Week 6: healing in 3 patients in group 1 (60%), in 4 of group 2 (80%) 1 in group 3 (20%) vs. 1 patient in the placebo group (16.7%). (P = 0.08 for group 2 vs placebo) • Week 12: healing in 2 patients in group 1 (40%), in 4 of group 2 (80%) 1 in group 3 (20%) vs. 2 patients sin the placebo group (33.3%) • Results confirmed by magnetic resonance imaging • Week 24: effects were maintained and even increased to 4 patients (80%) in group 1
Senjeoux 2015 [21]	Open clinical trial, multicentre randomized	54	Fistula plug as intervention against seton removal as control	1++; 8.658	• Fistula closure at week 12 was achieved in 31.5% of patients in the intervention group and 23.1% in control. The relative risk was not statistically significant, p = 0.19 • No differences in outcomes were found in patients with complex perianal fistulas, 33.3%, versus simple fistulas 30.8% in intervention; 15.4% and 25.6% in controls respectively, p = 0.45 • Results confirmed by magnetic resonance imaging • Regarding to safety, at week 12, 17 patients developed at least one AE in the group intervention versus 8 at the controls (p = 0.07) • The intervention showed no superiority over control
Park 2015 [22]	Multicentre open pilot clinical trial	6	Comparison of two doses of stem cells derived from allogenic adipose tissue	3; 1.095	• Patients were included sequentially in two dosing groups with 3 patients each. The first 3 patients (group 1) received 1 × 10^7^ cells/ml depending on the length and diameter of the fistula tract • After 4 weeks, when the safety of this dose was determined, the next 3 patients (group 2) received a higher dose of 3 × 10^7^ cells/ml • There was no Grade 3 or 4 AE or AE related to treatment • Two patients in group 1 achieved complete closure of the fistula in month 4 and month 6. A patient in group 2 achieved complete closure at 8 weeks • The closure remained until month 8 in 3 of those patients • Magnetic resonance imaging was performed to evaluate the reduction of the fistula and confirm that the tract lacked inflammation
Göttgens 2015 [23]	Pilot study	10	Fistulectomy with platelet-rich plasma	3; 2.721	• Half of the patients had previous fistula surgery Fistula healing was 70% at 12 months 95% CI (33–89) • One patient (10%) had a recurrence and in two (20%) fistula was persistent after treatment • One patient (10%) reported a persistent fistula and produced an abscess after the operation. No other complications were observed • In case of doubt about closure, magnetic resonance imaging was performed to visualize a possible fistula tract • The median in Vaizey’s incontinence severity scale score was 8.0 (range 0–21) indicating a moderate to severe continence impairment (0 perfect continence–24 total incontinence)
Cho 2013 [24]	Phase I clinical trial, open, dose escalation, multicentre	10 (safety assessment) and 9 (efficiency assessment)	Mother cells derived from adipose tissue	3; 3.341	• There were no Grade 3 or 4 related AE • Curation (complete fistula closure and internal and external openings without drainage or signs of inflammation) was obtained in 50% of the patients treated with at least 2 × 10^7^ cells/ml after a single injection
Lee 2013 [25]	Phase II clinical trial	43	Treatment of stem cells derived from autologous adipose tissue	3; 3.341	• Thirty-six patients completed the 8 weeks of follow-up after the final dose of stem cells. Of these, 33 formed the protocol analysis population • Complete fistula healing was observed in 27/33 patients (82%) after 8 weeks • Of 27 patients with fistula healing, 26 patients completed an additional observation study for 1 year and 23 patients (88%) kept the full closure • There were no AE related to stem cell administration • The treatment was well tolerated, with a favourable therapeutic result • The full closure was sustained
De la Portilla 2013 [26]	Phase I, open, multicenter, single-arm clinical trial	24	Mother cells derived from adipose tissue	3; 2.108	• The full analysis of efficacy data at week 24 showed 69.2% of the patients with a reduction in the number of draining fistulas, 56.3% of the patients achieved complete closure of the treated fistula achieved, and 30% of the cases presenting complete closure of all existing fistula tracts • Magnetic resonance images showed a reduction in week 24
Ciccocioppo 2011 [27]	Series of consecutive cases	12	Mesenchymal stem cells derived from bone marrow	3; 19.819	• Seven cases made complete closure • Three cases made partial closures • Results confirmed by magnetic resonance imaging • The disease improved in patients with reduced CDAI and PDAI indices • No AE were reported
Classic techniques					
Graf 2016 [28]	Retrospective observational study	119	Results after different surgical treatment with seton the most frequent	3; 1.095	• Of the 119 patients, 62 (52%) were healed at final follow-up. Fourteen healed after one procedure and the remaining 48 healed after a further median of 4.0 (2–20) procedures • Ten patients (8%) underwent a proctectomy • Healing was most common in patients operated with a procedure aimed at eradicating fistula (p = 0.0001), patients without proctitis (p = 0.02) and patients with a shorter duration of CD (p = 0.0019) • Results confirmed by magnetic resonance imaging
Gingold 2014 [29]	Prospective observational study	15	Fistula path intersphynterial ligation (LIFT technique)	3; 10.13	• At 2 months of follow-up, remission was observed in 9 patients (60%) without fecal incontinence • Clinical remission corresponded to an improvement in quality of life scales (Wexner and PDAI p = 0.001 and p = 0.0001 respectively) • At 12-month follow-up, only 33% (5/9) of patients who had remission maintained it
Galis-Rozen 2010 [30]	Retrospective review	77 17 (CD), 60 (non-CD)	Permanent loose seton in the treatment of high fistulas in patients with CD (29 procedures in total) and two-stage fistulotomy in patients without EC (107 procedures in total)	3; 2.769	• Early postoperative complications (within the first month) were observed in 8 patients (10%) (5 patients without CD, 3 with CD) • Perioperative complications, mainly local sepsis and bleeding, were recorded in 8 patients (10%) and long-term (> 1 month after operation), complications were observed in 9 patients without EC and 4 patients with EC • After 24 months (range 6–48) of follow-up, the recurrence rate was 40% in patients with CD and 47% in patients without CD • Five patients (4 patients without CD and one CD patient) developed some degree of faecal incontinence

*AE* adverse effects, *CD* Crohn disease, *CDAI* Crohn Disease Activity Index, *IF* impact factor; *MMC* mesenchymal mother cells, *PDAI* Perianal Disease Activity Index

**Table 2:** Results of the complex perianal fistulas of cryptoglandular origin analysis

Author and year	Design (as appears in the paper)	N	Intervention	Evidence level and IF	Main outcomes
Minimal invasive techniques					
De la Portilla 2019 [31]	Double-blind randomized parallel groups phase III study	56	Platelet-rich plasma vs. fibrin plug	1+; 2.108	• 32 patients with platelet-rich plasma and 24 with fibrin plug were treated • The healing rate was 71% in platelet-rich plasma versus 58.3% in patients with fibrin plug (p = 0.608) • The complete healing rate was 48.4% versus 41.7%, respectively; partial healing rate was 22.6% versus 16.7%, respectively • Patients receiving platelet-rich plasma had increased pain reduction, (p = 0.023) which caused an impact on improving quality of life in patients receiving platelet-rich plasma • Data from abstract congress, paper published in: Stem Cells Transl Med. 2020;9(3):295–301
Dozois 2019 [32]	Open phase I clinical trial	15	Mesenchymal stem cell-coated fistula plug derived from adipose tissue	3; 3.991	• Treatment-related short-term side effects were observed in 3 patients • At 6 months, 3 patients had complete healing, 8 partial healing and 4 showed no clinical improvement • Radiographic improvement was observed in 11 out of 15 patients. In 8 of these patients the fistula tract was smaller and reduced compared to baseline
Di Visconte 2018 [33]	Retrospective analysis	31	Injection of porcine collagen paste versus rectal feed with flap	2+; 2.108	• Five patients (16%) in the rectal feed group and one (5%) in the pig collagen group experienced fecal incontinence after the operation • Disease-free survival at age 2 was 65% in the rectal feed group and 52% in the pig collagen group (p = 0.659) • Average satisfaction scores were 5 (range 1–10) in the rectal feed group and 7 (range 2–10) in the pig collagen group (p = 0.299)
Marinello 2018 [34]	Retrospective observational study	10	OTSC device® (*over-the-scope-clip*)	3; 1.323	• Nine fistulas were of cryptoglandular origin and one patient had CD controlled with thiopurines • 80% of patients had previous anal surgeries • The healing rate of the fistula was 60% with an average follow-up of 15 months (range: 6–26 months) • Three patients had clinical recurrence and one patient required removal of the clip for invalidating pain • There was no appearance of fecal incontinence
Schniewind 2018 [35]	Pilot study based on prospective case series	7	Endoluminal vacuum therapy (VAC) with polyuretan sponge	3; 3.991	• Four patients had fistula of cryptoglandular origin and 3 related to CD • All patients tolerated therapy well and no AE were observed • The closure of the fistula tract occurred within 4 weeks of the completion of vacuum therapy • A patient with cryptoglandular fistula developed a recurrence within the 3-month follow-up
De la Portilla 2017 [36]	Phase II clinical trial	36	Autologous platelet growth factors	3; 2.571	• 7 AE related to injected product or surgical procedure was detected in 4/36 patients • 33.3% of patients (12/36) achieved complete fistula healing and 11.1% of patients (4/36) had partial healing • In successfully healing patients, a reduction in gradual pain was detected through an AVS, from 5.625 to 0.125 after 1 year, p = 0.0438 • A significant improvement in Wexner score was observed in patients achieving total or partial fistula healing, from 3.0625 to 1.125 in a year, p = 0.0195
Choi 2017 [37]	Phase I clinical trial	15	Stem cells derived from adipose tissue, two-dose comparison	2+; 2.721	• Nine out of 13 patients had fistula closure, 69.2% • Six days follow-up patients kept closing at 6 months, 83.3% • No AE were reported
Nordholm 2017 [38]	Retrospective unique cohort study	35	Nitinol clip	3; 3.991	• The healing rate of fistula 1 year after the procedure was 54.3% (19 out of 35 patients) • At the end of follow-up, 49% (17 out of 35) of patients had persistent closure of the fistula • No deterioration of continence was observed • The result of treatment was not found to be statistically associated with any clinical-pathological characteristics
Giordano 2016 [39]	Prospective, multicenter observational study	30	Collagen permacol paste	3; 1.095	• Of the 28 patients with available data, 15 patients (54%) presented a fully curated fistula at 6 and 12 months • More than 60% of patients were satisfied or very satisfied with the intervention
Ratto 2016 [40]	Estudio observacional prospectivo	10	Dispositivo Curaseal AF	3; 3.665	• Two months after the intervention, healing and absence of discharge or abscess formation was achieved in 5 patients (50%) • The final percentage of success was 70%, 7/10
Prosst 2015 [41]	Pilot study	20	OTSC device® (over-the-scope-clip)	3; 1.095	• Within 6 months of surgery, 18 patients (90%) were considered cured because they had no clinical signs or symptoms of fistula • In 13 of the 18 patients (72%), the clip caused no problems, while in 3 patients the clip spontaneously detached. In the remaining 2 patients the clip was removed due to discomfort and late wound healing • There were no AE related to the clip, such as necrosis or ischemia pressure ulcers • No case of fecal incontinence was observed during follow-up
Borowski 2015 [42]	Prospective one-arm study	7	Treatment with stem cells derived from autologous adipose tissue	3; 1.33	• 71.4% of patients showed signs of fistula closure • 57.1% had complete fistula closure at 46 months (average) follow-up • There were no AE associated with the technique • There were no new cases of incontinence
Stamos 2015 [43]	Prospective observational study	93	Biosabsorbable synthetic anal fistula plug	3; 4.087	• Fistula healing rates at 6 and 12 months were 41% (95% CI 30–52%; total, n = 74) and 49% (95% CI 38–61%; total n = 73), respectively. Half of the patients in which prior treatment failed achieved healing • At 6 months, Wexner’s average score improved significantly (p = 0.0003) • At 12 months, 93% of patients had minimal or no pain • AE included 11 infections/abscesses, 2 new fistulas and 8 total and 5 partial plug extrusions • Fistula healed in 3 patients with partial extrusion
Ozturk 2014 [44]	Pilot study	10	Autologous cartilage plug	3; 2.721	• Nine patients had cryptoglandular abscess, and one patient had Crohn’s disease • The average follow-up time was 24 months (range 10–32 months). Of the ten patients, nine were treated for fistula without complications in the short term • Two late recurrences were observed among the nine patients with successful operations
Tan 2013 [45]	Retrospective observational study	30 anal fistula plugs in 26 patients	Anal fistula plug	3; 1.095	• Twenty-nine (96.7%) fistulas had a previously inserted seton with an average duration of 12 weeks before the anal fistula stopper procedure • After a follow-up of 59 weeks (median) (range 13–97) 26 (86.7%) fistulas resorted • Of these 26 cases, the time to failure was 8 weeks (range 2–54). Surgical interventions were needed in 20 of the 26 cases
Almeida 2013 [46]	Retrospective observational study	51 (41 with complete follow up)	Biological anal fistula plug	3; –	• Twenty-three (56.1%) patients had complete healing while 18 (43.9%) patients failed the fistula plug procedure during the follow up period of 12 months
Heydari 2013 [47]	Retrospective review	48 patients with 49 fistulas	Fistula plug made with biosorbable polymers	3; 3.991	• The overall healing rate in the series was 69.3% (34/49 fistulas, 33/48 patients) • Eight patients (24.2%) had fistula healing within 3 months after surgery, 21 patients (63.6%) 6 months and 4 patients (12.1%) 12 months • At 3 months, no patient had perineal pain or fecal incontinence
Herreros 2012 [48]	Randomized, simple blind clinical trial	200	Treatment with stem cells derived from autologous adipose tissue	1+; 3.991	• Group A: 64 patients; 20 million stem cells; Group B: 60 patients, 20 million stem cells derived from adipose tissue plus fibrin glue; Group C: 59 patients; fibrin glue • The intention to treat population comprised 183 patients out of 200. Of these, 165 (90.2%) completed the study • The healing rates of fistula at week 12 were 26.56%, 38.33% and 15.25% in arms A, B and C, respectively (p = 0.01) • A total of 61.75% (n = 113) patients received 2 doses of treatment. The healing of the fistula after the second dose was 39.1% (n = 25), 43.30% (n = 26) and 37.29% (n = 22) (p = 0.79). The healing time was similar in all groups • The proportion of patients with fistula healed at week 12 who experienced reopening at week 24 to 26 was 25.00%, 14.29% and 11.11% (p > 0,5). These patients did not receive a second dose of treatment • No statistically significant differences were found when comparing SF-36 (mental and physical health domains) at baseline and at 24 to 26 weeks • Most patients suffered at least 1 AE (proctalgia, pain, perianal abscess, itching, swelling); 59 AE (90.8%), 49 (84.5%) 51 AE (85.0%) groups A, B and C, respectively (p = 0.51) • Each year, the healing rates were 57.1%, 52.4% and 37.3% in groups A, B and C, respectively (p = 0.13)
Ommer 2012 [49]	Retrospective observational study	40	Synthetic anal fistula cap (Gore BioA Fistula Plug®)	3; 1.17	• Six months after surgery, fistula healed in 20 patients (50%). Three fistulas healed after 7, 9 and 12 months • The overall healing rate was 57.5%, dependent on the number of previous interventions • In patients having only drainage of the abscess, success occurred in 63.6% (14/22) whereas in patients after one or more flap fistula reconstruction, the healing rate decreased slightly to 50% (9/18)
Cintron 2012 [50]	Prospective follow-up study	72, 11 with CD	Inserting a swine intestinal submucosa plug	3; 2.635	• There was no difference in closing rates between primary and recurring fistula, 38% and 40%, respectively • The overall patient success rate was 38% • Patients with Crohn’s disease had a 50% closure (4/8) • There were no intraoperative complications • Four postoperative abscesses (4/73; 5) were reported
Wilhem 2011 [51]	Pilot study	11	Use of the FiLaCLaser ® in fistula repair	3; 2.721	• Nine out of eleven patients had primary healing (81.8%) • No side effects were reported during follow-up
Van der Hagen 2011 [52]	Prospective randomized study	15	Mucous feed cap vs. fibrin sellant	1+; 1.806	• Three (20%) patients in the mucosal feed plug group had a recurrent fistula compared to 9 (60%) of the fibrin sellant group, (p = 0.03) • No differences were reported between groups in quality of life or incontinence score
Van der Hagen 2011 [53]	Pilot study	10	Platelet-rich autologous plasma	3; 1.095	• One patient (10%) presented a recurring fistula after 12 months • Patients did not develop de novo incontinence • No adverse events were reported • Platelet-rich plasma may be a valid alternative in patients with cryptoglandular fistula
El-Gazzaz 2010 [54]	Retrospective review of cases	33	Biologically absorbable anal fistula plug	3; 1.095	• Thirty-three patients underwent 49 plug inserts 61% of fistulas were of cryptoglandular origin and 39% related to CD • Eight out of 32 patients (25%) (1 patient was loss of follow-up) had successful closure on all of their fistulas • The success rate after the first intervention was 8/33 patients (24.2%) • Fourteen patients underwent a second plug insertion, 2 patients (14.3% succeeded in closing the fistula) • Two patients underwent a third attempt at closure with plug insertion; one had fistula closure • The success rate for cryptogenic fistulas was 34.6% (9/26) vs 9.1% (2–22) of CD-related fistulas • Closure was most successful in patients with the placement of a seton prior to the insertion of the plug, 28 vs 4 patients without seton, p = 0.05 • Fistula recurrence was observed in 24 patients (75%) with an average recurrence time of 56 days (range 7–251 days). Two patients had late recurrence at 6 and 8 months after insertion of the plug
Lenisa 2010 [55]	Prospective observational study	60	Collagen plug	3	• Thirty-eight fistulas were recurrent • The average operating time was 26 ± 10 min • There were no serious complications, no sepsis, no cases of mortality • The average recurrence time was 5.7 ± 1.7 months. Recurrences were observed in 24 patients, resulting in a rate of 60% success per patient • The average reduction in the score in the continence rating was 0.6, 95% CI (1.3– −0.1); p = 0.01 • The overall success rate of recurrent patients was 72% without impaired continence
Owen 2010 [56]	Retrospective observational study	32 patients with a total of 35 insertions	Cook Surgisis AFP Plug™	3; 1.355	• The overall healing rate at the end of the follow-up period (15 months; range 2–29 months) was 37% (13 out of 35 patients) • By fistula type, the healing rate of CD-related fistulas (1 out of 3) was 33% and (11 out of 31), 35%, in cryptoglandular origin fistulas
Queralto 2010 [57]	Prospective observational study	34	Synthetic glue	3; 1.14	• The healing rate in the first month was 67.6% (23 patients); the fistula was unable to close in 11 patients • Of the 23 patients with cured fistula, all remained free of recurrence, with no disorders associated to continence during the 34 months of follow-up (median) (range 21–43 months)
McGee 2010 [58]	Prospective observational study	41 patients with 42 fistula tracts	Anal fistula plug in fistula tracts > 4 cm	3; 3.991	• The complete healing was achieved in 18 out of 42 (43%) fistulas over an average follow-up period of 24.5 months • There were no AEs or cases of incontinence during postoperative clinical visits • The successful closure of the fistula was significantly associated with a longer length of the tract; fistulas larger than 4 cm were nearly threefold more likely to heal compared to shorter fistulas
Classic techniques					
El-Said 2019 [59]	Pilot study	32	Modification of the original Park technique extending the internal sphincterotomy	3; 1.841	• Two patients (6.25%) experienced recurrence and 5 (15.6%) developed complications (urinary retention, moderate postoperative bleeding, wound infection and new onset fecal incontinence (1 patient) • Twenty-eight (87.5%) patients were completely satisfied with the procedure • Quality of life showed a significant improvement at 6 months. All the physical and mental components of the SF-36 questionnaire showed a significant increase, except in the role of limitations due to emotional problems that showed a non-significant increase (p = 0.7)
Dadou 2018 [60]	Retrospective case series study in a single center	76	Drainage seton	3; 3.991	• The average time for seton removal was 36.6 weeks (range, 6.0–188.0 weeks) • The average follow-up was 63 months (range, 7–121 months) • Fifty-six patients (73.7%) had full resolution of symptoms and 14 (18.4%) had a significant improvement in symptoms without the need for additional surgical treatment • Six patients (7.9%) had persistent severe symptoms and five (7.1%) had a recurrence after seton removal
Podetta 2018 [61]	Retrospective observational study	32	Mucosal advancement flap	3; 2.108	• Of the 121 patients (group A) treated with flap, 32 (26.4%) (group B) lodged appeals and required a second procedure • Group B healing rate was 78.1%. Six patients in this group needed a second surgery with healing in all cases • The complication rate was 9.4% in group B compared to 8.3% for patients in group A • A slight continence deficit (Miller score 1, 2 and 4) was detected after the first forward flap in 3 patients. Miller’s score did not change after the next procedure
Balciscueta 2017 [62]	Retrospective observational study	119	Change in resting pressure after full thickness rectal feed flap	3; 2.125	• Significant decrease in maximum postoperative resting pressure was reported (from 90.6 ± 31.9 to 45.2 ± 20 mmHg; p < 0.001) • The minimum pressure values did not differ significantly (from 8.2 ± to 18.3 to 23.2 ± 13.5 mmHg; p = 0.1) • Recurrence rate: 5.9% • Same percentage of patients with anal continence before and after (76.5%)
Boenicke 2017 [63]	Prospective observational study	61	Flap technique	3; 2.234	• The independent parameters for the failure of therapy in a multivariate data analysis were history of surgical drainage of abscesses [OR = 8.09, p = 0.048, 95% CI (0.98–64.96)], supraspherical fistula [OR = 6.83, p = 0.032, 95% CI (1.17–6.83)] and BMI [OR = 1.23, p = 0.017, 95% CI (1.03–1.46)] • Low risk of incontinence
Emile 2017 [64]	Case control retrospective study	251	Seton placement	2+; 1.84	Recurrences were observed in 26 patients (10.3%) after a mean duration of 12.2 ± 3.9 months after removal of seton Previous recurrent fistulas (OR = 2.81, p = 0.02); supra-lift extension (OR = 3.19, p = 0.01), previous fistulas (OR = 3.36, p = 0.004) and horseshoe fistula (OR = 5.66, p = 0.009) were the most significant predictors of recurrence Cases of fecal incontinence were detected as complications in 8 patients (3.2%) and seton infection in 16 patients (6.3%) The female sex (OR = 15.2, p = 0.0003) and the horseshoe fistula (OR = 8.66, p = 0.01) were significant risk factors for fecal incontinence following the procedure
Sugrue 2017 [65]	Retrospective review	462	Sphincter-sparing repair	3; 3.991	• 420 sphincter-sparing repairs (44%) resulted in healing and 283 (56%) resulted in non-healing with a median follow up of 9 (range, 1–125) months • The median time to recurrence of fistula was 3 months (range, 0–75) with 79% and 91% of recurrences observed within 6 and 12 months. Patients treated with dermal feed flap, rectal feed flap or ligature of the intersphynteric tract procedure were less likely to have a recurrence than patients treated with a fistula plug or fibrin glue (p < 0.001)
Herold 2016 [66]	Prospective and multicenter observational study	60	Synthetic Plug (GORE® BIO-A®)	3; 2.721	• Complete follow-up (12 months) of all the patients was reached • Mean intervention time was 32 ± 10.2 min and mean duration of the hospital stay was 3.3 ± 1.8 days • No intraoperative complications were observed in any patient. The healing rate after 4 weeks was 6% (3 out of 54 patients), and after 3 months it was 42% (18 out of 46 patients). The healing rate after 6 months of follow-up did not change and stayed just above 50% • Treatment had no consequences on continence • The rate of displacement of the plug was 10% (6 out of 60 patients) at 6 months of the operation • 34% of patients (16 out of 47) required a new operation
Seow-en 2016 [67]	Retrospective study of consecutive patients	41	Assisted video treatment	3; 2.721	• Primary healing rate was 70.7% with a median follow-up of 34 months • Twelve patients resorted or did not heal and underwent a repeated procedure • The secondary healing rate was 83% with two recurrences • Overall, the stapling of the internal opening had a 22% recurrence rate, while the anorectal feed plug had a failure rate of 75% • No recurrence was observed in six cases after using the over-the-scape clip
Visscher 2016 [68]	Retrospective study	143	Fistulotomy and fistulectomy combined with mucous flap	2+; 1.095	• 27% of patients had recurrence • The risk factors for recurrence were secondary pathway formation [HR: 2.4; 95% CI (1.2–51), p = 0.016] and previous fistula surgery [HR: 1.2; 95% CI (1.0–4.6), p = 0.041]
Raslan 2016 [69]	Prospective descriptive study	51	Cutting seton	3; 0.808	• Recurrence rate was 9.8% • Postoperative incontinence rate was 15.7% for gases and 5.9% for liquid depositions. There was no solid stool incontinence • The cutting seton is a valid option for complex perianal fistulas, but in patients and previous perianal surgery, other surgical options are recommended
Rosen 2015 [70]	Retrospective review	121	Cutting seton	3; 1.095	• The median time to healing was 3 months (range 1–18) • 7.4% of patients required additional surgery but 98% had a complete fistula healing • The incontinence rate decreased from 19 to 11.6% postoperatively • Of 23 patients with pre-intervention incontinence, 17 (73.9%) resolved his symptoms
Soliman 2015 [71]	Case series study	140	Cutting seton	3; 5.238	• Most patients, 111 (79.3%), had cryptoglandular fistulas and 14 patients (10%) had CD-related fistulas • Of the 111 patients, 81 (73.0%) presented transsphynteric fistulas. After 35 months of follow-up (mean) (range, 2–83 months), 70 transsphynteric fistulas had healed (86.4%), 10 were still being treated (12.3%) and one patient was loss of follow-up (1.2%) • Six patients developed recurrence (7.4%). Three “true” recurrences (3.7%) and three “de novo” fistulas (3.7%) • No cases of incontinence were reported
Lee 2015 [72]	Retrospective review	61	Advancement flap	3; 2.387	• Fifty-three (86.9%) surgeries developed successfully, while in 8 of them (13.1%) the procedure failed. Four of them underwent further surgery • Of the 53 patients who had a successful procedure, 27 responded on the Wexner scale, 21 patients (77.8%) presented a score of 0 (perfect continence)
Uribe 2015 [73]	Retrospective observational study	119	Tunneling fistulectomy (core out) and curettage	3; 2.387	• The “Core out” technique was performed in 78 patients (group I) and curettage in 41 (group II) • The total recurrence rate was 5.88%, 5 of group I (6.4%) and 2 of group II (4.9%), without statistical significance (p = 0.74)
Gottgens 2015 [74]	Retrospective observational study	537	Fistulotomy	3; 2.387	• 88 patients (16.4%) had recurrence which resulted in a primary healing rate of 83.6% • Of the 88 patients with recurrence 40 healed, resulting in a secondary healing rate of 90.3% • Kaplan–Meier’s analysis showed that 1 year healing rate was 0.70, 95% CI (0.33–0.89) • Major incontinence defined as a Vaizey mean value greater than 6 was reported in 28% of patients; the average score was 4.67 (SD: 4.80) • Only 26.3% of patients had a perfect continence state (Vaizey = 0) • The SF-36 survey scores were no different from the general population • It is not clearly specified whether they are simple or complex perianal fistulas, which can condition the results, the author refers that they are of high type
Patton 2015 [75]	Retrospective observational study	59	Cutting seton	3; 1.355	• Mean follow-up time was 9.4 years (range 1.7–15.6 years) • The majority of patients had a single seton (n = 56) and three patients had two setons • Mean time from seton insertion to the time of follow-up where healing was noted (primary healing) was 17.7 months (median 11 months). Four patients (6.8%) developed recurrent fistulas. Three of the four patients underwent a second cut-off seton treatment, the fourth continuing to be treated • The primary healing rate was 93% (55 cured) and the secondary rate was 98% (58) • Seventy-eight percent of patients had normal continence or minor incontinence (St. Mark score 0–6), 13.5% moderate incontinence (score 7–12) and severe incontinence 8.5% (score > 12). 63% of patients had no changes or improved control • St Mark’s continence scores showed a reverse correlation with FIQL (p < 0.001). Average FIQL scores were high and correlated significantly with continence • The average patient satisfaction score was 9 out of 10
Hirschburger 2014 [76]	Retrospective observational study	50	Fistulectomy with primary sphincter reconstruction	3; 2.387	• Fistula healing was obtained in 44 patients (88%) who, moreover, did not develop recurrence • In 5 patients (10%), fistula healed, but they developed a recurrence during the observation period (average follow-up 22 months). In 1 of these patients (2%) the fistula didn’t close • The score on the continence scale before and after the operation showed a slight decrease in continence in 3 patients. A patient with 2nd grade incontinence improved • Pre-existing pain was significantly reduced with the intervention
Ratto 2013 [77]	Prospective observational study	72	Fistulotomy after primary sphinteroplasty	3; 3.991	• Of the 72 patients, 12 (16.7%) had fistula recurrences and 29 patients (40.3%) required seton drainage following surgery • Three patients had recurrence • Eight patients (11.6%) without basal incontinence) reported spotting after defecation • No factors related to surgical success were located • Patients with recurrent fistula after previous surgery were five times more likely to be affected in continence
Van Onkelen 2013 [78]	Case series	14	Reparation combined with abscess drainage	3; 3.991	• Healing was reported in 79% patients • The 3 patients who were not cured at the first intervention were given a second, third or fourth intervention with 100% healing • The median Rockwood Fecal Incontinence Severity Index incontinence score after the intervention was 0
Wallin 2012 [79]	Retrospective review	93	Ligation of the intersphincteric fistula	3; 3.991	• The median follow-up time was 19 months (range, 4–55) • Thirty-two patients (32%) had a history of previous surgery • The healing success rate was 40% after the first ligature procedure • The total success rate after ligature including patients previously treated with ligation of the intersphincteric fistula was 47% (44 out of 93) • Patients with successful fistula closure reported an average CCFFIS score of 1.0 (± 2.3) • No predictors were found for the successful closure of the fistula
Abbas 2011 [80]	Retrospective review	169	Fistulotomy, advancement flap, and fistula plugging	2+; 13.265	• Failure of intervention: 15.6%, 15.6% and 7.3%, in fistulotomy, feed graft and fistula capping respectively • The plugging had the highest failure rate (83.3%) compared to fistulotomy (10.1%) [OR: 44.3; 95 CI (8.9–221.0), p = 0.001] • Transphincteric and suprasphinteric high fistulas were incontinence predictors compared to subcutaneous fistulas with OR of 22.9, 95% CI (2.2–242.0), p = 0.009] and 61.5; 95% CI (4.5–844.0), p = 0.002), respectively • The only predictor of septic complications was plugging compared to fistulotomy [OR: 15.1; 95% CI (2.3–97.7), p = 0.004]
Mitalas 2010 [81]	Retrospective observational study	278	Seton drainage prior to transanal advancement flap	3; 2.108	• The average healing time was 2.2 months • In patients without preoperative seton drainage, the healing rate was 63%, whereas the healing rate was 67% in patients who underwent preoperative seton drainage • The overall healing rate was 64% • The preoperative drainage seton did not improve the result of repair with forward flap
Roig 2010 [82]	Retrospective study	146	Endoanal advanced graft (Group A, n = 71) vs. immediate sphincter repair after fistulectomy (Group B, n = 75)	2+; 2.769	• After a mean follow up of 13 months (12–60), fistula persisted or recurred in 13 (18.3%) patients in Group A vs 8 (10.6%) in Group B (p = 0.19). Thirty-one (43.6%) patients in Group A vs 16 (21.3%) in Group B presented postoperative continence disturbances (p < 0.001) • The average postoperative stay (SD) was 6.9 (2.4) days in Group A versus 5.9 (2.5) in Group B (p = 0.01) • No changes were observed with the FIQL scale • Group A patients had a significant reduction in maximum resting pressure after surgery

*AE* adverse effects, *CD* Crohn disease, *CDAI* Crohn Disease Activity Index, *CI* confidence interval, *HR* hazard ratio, *FIQL* Fecal Incontinence Quality of Life, *IF* impact factor; *OR* odds ratio, *MMC* mesenchymal mother cells, *PDAI* Perianal Disease Activity Index, *SD* standard deviation

We found that the concepts of healing and fecal incontinence were defined differently in the various studies, making quantitative aggregation difficult; therefore, these issues must be considered when interpreting our results. Nevertheless, we were still able to obtain relevant information from the review.

### Clinical outcomes

Of the 18 articles with clinical results that referred to CD, 4 reported level 1 evidence, one reported level 2 evidence, and 10 reported level 3 evidence. A total of 15 articles referred to MIS techniques, and three referred to classical techniques. Studies referring to the most current techniques were more common than those referring to traditional techniques, the evidence levels presented for the current techniques were higher, and the results were more favorable. Two studies employing treatments using derivatives of adipose tissue reported healing rates exceeding 70% [15, 25], and a significantly greater proportion of patients stem cells-treated achieved combined remission versus controls (56.3% vs 38.6%, p = 0.010) in a high-level evidence study [14]. In a study investigating a treatment using mesenchymal cells, in which healing was defined as fistula absence or less than 2 cm discharge on magnetic resonance imaging, the authors reported a healing rate of 80% at week 12 [20]. In a pilot study with 10 patients undergoing fistulectomies with platelet-rich plasma, one (10%) patient experienced a recurrence, and two (20%) patients had persistent fistulas after treatment. In two studies examining classical techniques (primarily seton, LIFT, or lay open), the healing rates were approximately 50–60% [28, 29]. Graf et al. observed that 62 (52%) patients achieved healing (absence of fistula symptoms, skin healing, and no evidence of a fistula on clinical examination) by the end of the follow-up period, but only 14 of the patients had healed after a single procedure, while the remaining 48 healed after a median of 4.0 (2–20) additional procedures [28].

Of the 52 references referring to clinical results for patients with fistulas of cryptoglandular origin, three of the articles reported level 1 evidence, six reported level 2 evidence, and 43 reported level 3 evidence. A total of 28 articles referred to MIS (25 articles reported the use of plugs), and 24 articles referred to classic techniques (six articles reported the use of seton, and four studies incorporated flaps). The MIS studies reported healing rates between 50% and 90%; a healing rate of 70% was reported for a study using derivatives of adipose tissue [42], an 80% healing rate was achieved using laser technology [51], a 70% healing rate was achieved using platelets [31], and healing rates ​between 50% and 90% were achieved using various plugs [34, 38, 41]. Additionally, each of these studies presented results for various other aspects of complex perianal fistulas, such as pain, quality of life, or continence; continence was most frequently reported in these studies, and improved results were typical. Decreases in Wexner scores after the use of autologous platelet growth factors [36] and Nitinol Clips [38] were also reported. No fecal incontinence was reported after procedures performed with over-the-scope-clips [41] or stem cells derived from autologous adipose tissue [42]. Recurrence and retreatment occurred in 2/10 cases [44] and 20/25 cases [45] using two kinds of plugs.

For some studies that incorporated classic techniques, the healing percentages were similar to those observed in the MIS studies (70–80% for setons [60] or flaps [61] and somewhat lower percentages for other techniques, such as a fistulotomies or fistulectomies [23, 40]); however, the overall function in these patients was lower, as more cases of incontinence were reported, either because the incontinence was not corrected or it appeared de novo. In a study investigating fistulotomies, only 26.3% of the patients had a perfect continence state, with a Vaizey score equal to 0 [74]. The recurrence or retreatment rates in these studies varied from 5.9 to 50% [62, 66, 67].

### HRQoL outcomes

Four HRQoL studies in this analysis were performed for CD, but only one of them showed a relationship between the results and the surgical technique used. In a post-hoc analysis of the ADMIRE-CD clinical trial, Panes et al. [83] observed that patients who experienced clinical or combined remission had lower (Perianal Disease Activity Index) PDAI scores for pain and discharge than those who did not experience remission. The scores were fourfold higher for patients who experienced clinical or combined remission in combination with magnetic resonance imaging. The scores were fourfold higher for patients who experienced clinical or combined remission in combination with magnetic resonance imaging. Other HRQoL studies referred to abdominal surgery [84] or perianal disease [85, 86] in general, so they did not refer to surgery.

Three studies with HRQoL outcomes for patients with cryptoglandular CPF were included in this review. Jayne et al. [87] compared the efficacy of the Surgisis anal fistula plug with various other techniques in a prospective, multicenter, randomized, unblinded, parallel arm clinical trial. A total of 304 patients were included in their study, and the authors observed no differences in the clinical healing rates (55%, 64%, 75%, 53%, and 42% for the fistula plug, seton cut, fistulotomy, advancement flap, and LIFT procedure, respectively) at 12 months. The baseline fecal incontinence rates were lower for the groups with little improvement after treatment. The mean total costs were £2738 (± £1.151) for the fistula plug group and £2308 (± £1.228) for the surgeon’s preference group. The Quality Adjusted Life Years (QALYs) were higher for the fistula plug group (0.829 ± 0.174) than for the surgeon’s preference group (0.790 ± 0.212), which establishes that there is a 35–45% chance that the fistula plug is as profitable as the surgeon’s preference for an availability to pay range of £20,000–30,000/QALY.

In a prospective study of 34 patients undergoing surgical treatment, Jayarajah [88] reported overall preoperative and postoperative incontinence rates of 18% and 38%, respectively. The total mean Fecal Incontinence Quality of Life (FIQL) score was 16.0 (Standard Deviation, SD ± 0.4) preoperatively and 16.1 (SD ± 0.4) postoperatively. The authors also observed a considerable difference in the scale that measures “depression/self-perception” before and after the intervention (p = 0.012). In a retrospective cross-sectional study, Visscher et al. [89] found that by the end of the follow-up period (mean follow-up of 7.8 years) 39/141 patients (34%) who underwent unspecified surgical procedures after an initial perianal fistula surgery still experienced incontinence. Surgical fistulotomies, drainage of multiple abscesses, and high transsphincteric or suprasphincteric abscesses were associated with incontinence to a significant degree. Incontinence was worse for patients who had surgery for CPF (Wexner score, 4.7 ± 6.2) than for those who had surgery for simple fistulas (Wexner score, 1.2 ± 2.1) (p = 0.001). Surgery for CPF was also associated with worse quality of life outcomes, including lifestyle (p = 0.030), depression (p = 0.077), and shame (p < 0.001).

### Cost outcomes

Of the articles included in this review, only the Jayne et al. article [87] associated a technique with its economic cost. In this study, a mean total cost was associated with the group who underwent treatment with fistula plugs (£2738 ± £1151) and the rest of the treatments (£2308 ± £1228). Additionally, a QALY gain of 0.829 ± 0.174 was calculated for the group with fistula plugs compared to 0.790 ± 0.212 for the surgeon’s preference group (0.790 ± 0.212). The probabilistic incremental cost-effectiveness results were £10,993 (± £478,666), with a 35–45% chance that the fistula plug is as profitable as the surgeon’s preference for an availability-to-pay range of £20,000–30,000/QALY. Three studies showed the cost of CD, but none showed the cost of any surgical techniques [90–92].

## Discussion

We conducted a comprehensive systematic literature review to audit the clinical outcomes resulting from surgical treatment of complex perianal fistulas. Various databases and sources of information were analyzed to determine the similarities between surgical treatments for CD-associated fistulas and cryptoglandular complex perianal fistulas. Studies by Graf [28], Gingold [29], and Galis-Rozen [30] showed that classic techniques, such as lay open, LIFT, fistulectomies, and flaps, are still used to treat CD, despite the potential risk of exposure to the anal sphincter. The communicated healing percentage for CD was approximately 50% usually after the repeated procedures [28, 30], and the percentage of patients with incontinence rose to 60% (9/15) by the end of the follow-up period [29]. These results were surpassed decisively by those for MIS, a difference of 17% in remission rates was reported in stem cells-treated patients in a clinical trial [14] and healing rates close to 80%, with a reduction in the PDAI and improvements to the HRQoL in some patient series’ [13]. In a study with autologous adipose-derived stromal vascular fraction a significant reduction in the severity of perianal disease was shown, with PDAI reduction from 7.3 to 3.4 in week 48 (p = 0.045). According to Tozer et al. [93] we hypothesize that the inflammatory origin present in the CD fistula prevents its complete healing with conventional techniques. Therefore, the use of cellular mechanisms with anti-inflammatory potential may have a favorable result in healing and maintaining sphincter function beyond the use of a single conventional technique. However, these conclusions must be ratified in subsequent studies, since the studies published up to the date of the review sometimes corresponded to series with few patients and a short follow-up period.

In CPF results on healing rate were similar between MIS and classic techniques. In MIS we found that in a study with OTSC device® (over-the-scope-clip) [34] there was no appearance of fecal incontinence, an improvement of Wexner score with the use of autologous platelet growth factors, from 3.0625 to 1.125 in a year, p = 0.0195 [35]; no deterioration of continence was observed with Nitinol Clip [38] as a result of the last 5 years. In classic techniques we found a study with a slight continence deficit in patients treated with Mucosal advancement flap [61], no improvement in continence was reported by Balciscueta et al. [62], and cases of fecal incontinence were detected as complications in 8 patients treated with seton [64].

We observed that during the 10-year period from 02/01/2010 to 02/29/2020, a shift occurred in the treatment of cryptoglandular CPF from classic surgical techniques to a MIS/biological approach; this shift has allowed better facilitation of the healing and preservation of sphincter function. Of the 18 papers that referred to CD, 13 reported on investigations regarding MIS, and four reported on classic techniques. We found that there was not a significant difference between the number of articles for each of the procedures, as there were 27 articles referring to MIS and 22 referring to classic techniques. Therefore, we suggest that a paradigm shift is beginning to occur, making MIS a first order treatment for CPF of cryptoglandular origin.

Notably, procedures like LIFT are recommended in some guidelines as a first option for patients with CD [94], although this is not supported by substantial evidence, as we found only one reference that advocated for this recommendation [29]. Therefore, we suggest that these recommendations should be reevaluated. Given what has been published, we believe that it is safer not to divide any part of the anal sphincter when treating perianal CD.

A critical point in the topic we are dealing with is the use of new pharmacological treatments such as anti-TNF for the management of patients with CD fistula. Although the objective of our study was to analyze the surgical techniques and pharmacological treatments that were excluded, we believe that it is appropriate to highlight this aspect. Treatment with infliximab indeed has good results in these patients, but it is also true that after 1 year of treatment the response rate can fall to 23% [95]. These results have recently been improved with the use of mesenchymal cells with annual response rates of 59% [96].

Nevertheless, these initial observations must be analyzed with consideration for the following limitations: First, our preliminary hypothesis was that the level of evidence from the selected studies could be low, and this would create a high risk of bias. The selected articles have confirmed this hypothesis, especially in the case of the articles about classic procedures. This made it difficult to carry out a more rigorous comparison of the results, such as meta-analysis or network meta-analysis. The different ways to present healing and continence results also made it difficult to aggregate the results.

We determined that there are no published studies that have specifically investigated the relationship between surgical techniques and quality of life. Therefore, we suggest that studies capable of determining the impact of the various surgical procedures from the patient’s perspective should be designed, as the HRQoL is only a secondary or tertiary variable in currently published studies. Only 6 of the 80 (7.5%) total references (Serrero, both in 2017 [15] and 2019 [13], Gingold [29], El-Said [59], Gottgens [74], and Herreros [48]) reported quality of life results. Consequently, we encourage the development of robust quality of life and cost-effectiveness studies, as both these variables are factored into our conclusions.

In conclusion, our review shows that patients with CD experience a higher rate of healing after MIS techniques than patients who undergo classic surgical techniques, and the healing rate for complex anal fistulas with cryptoglandular origins appears similar between classic and minimally invasive techniques. Additionally, the incontinence rate for patients that undergo minimally invasive surgical techniques is better than that of patients who undergo classic techniques. Therefore, we recommend moving to MIS-based techniques, in conjunction with new biological technologies like stem cells, plugs or Adipose-Derived Stromal Vascular Fraction use, because these techniques seem to be supported by recently published clinical evidence.

## Supplementary Information

**Additional file 1.** PRISMA checklist. The results of PRISMA checklist are shown in the next tables.

**Additional file 2.** Search strategy. The details of the search are listed in the next table.

## Data Availability

The search strategy is shown in Additional file 2, results of the search are stored in PORIB.

## Abbreviations

AE: Adverse Effects
AGA: American Gastroenterological Association
CD: Crohn’s Disease
CDAI: Crohn Disease Activity Index
CENTRAL: Cochrane Central Register of Controlled Trials
CI: Confidence Interval
CPF: Complex Perianal Fistulas
DARE: Database of Abstracts of Reviews of Effectiveness
ECCO: European Crohn’s and Colitis Organisation
ESCP: European Society of Coloproctology
FIQL: Fecal Incontinence Quality of Life
HR: Hazard Ratio
HRQoL: Health-Related Quality of Life
IF: Impact Factor
JCR: Journal Citation Reports
LIFT: Ligation of Intersphincteric Fistula Tracks
MIS: Minimally Invasive surgery
MMC: Mesenchymal Mother Ccells
OR: Odds Ratio
PDAI: Perianal Disease Activity Index
PICO: Participants, Interventions, Comparisons, Outcomes, and Study Design
QALYs: Quality Adjusted Life Years
RCT: Randomized Controlled Trials
SD: Standard Deviation
SIGN: Scottish Intercollegiate Guidelines Network
UEG: United European Gastroenterology
