# Supplementary material for: Surgical management of complex perianal fistula revisited in a systematic review: a critical view of available scientific evidence
Source: BMC Surg. 2023 Feb 5;23:29. doi: 10.1186/s12893-023-01912-z (PMC9901165; doi:10.1186/s12893-023-01912-z)
Supplement: Supplementary file 2 — Additional file 2. Search strategy. The details of the search are listed in the next table. [file 12893_2023_1912_MOESM2_ESM.docx]

Additional file 2: Search strategy

The details of the search are listed in the next table.

| # | Terms | Strategy |
| --- | --- | --- |
| #1 | Crohn disease | (("Crohn Disease"[Mesh]) OR "Crohn Disease") OR "Crohn's Disease" |
| #2 | Rectal fistula, perianal, Fistulizing disease, complex | ((((((("Rectal Fistula"[Mesh]) OR "perianal fistula") OR "perianal fistula*") OR "complex anal fistula*") OR "complex perianal fistula*") OR "Perianal disease") OR "fistulizing disease") OR "Fistula-in-ano" |
| #3 | #1 AND #2 | ((((((((("Rectal Fistula"[Mesh]) OR "perianal fistula") OR "perianal fistula*") OR "complex anal fistula*") OR "complex perianal fistula*") OR "Perianal disease") OR "fistulizing disease") OR "Fistula-in-ano")) AND ((("Crohn Disease"[Mesh]) OR "Crohn Disease") OR "Crohn's Disease") |
| #4 | Inflammatory Bowel Disease, Crohn Disease. | (((("Inflammatory Bowel Diseases"[Mesh]) OR "Inflammatory Bowel Disease*") OR "IBD")) OR ((("Crohn Disease"[Mesh]) OR "Crohn Disease") OR "Crohn's Disease") |
| #5 | Cryptoglandular, Rectal fistula, complex | ((((((((("Rectal Fistula"[Mesh]) OR "perianal fistula") OR "perianal fistula*") OR "complex anal fistula*") OR "complex perianal fistula*") OR "Perianal disease") OR "fistulizing disease") OR "Fistula-in-ano")) AND "Cryptoglandular" |
| #6 | #5 NOT #4 | (((((((((((("Rectal Fistula"[Mesh]) OR "perianal fistula") OR "perianal fistula*") OR "complex anal fistula*") OR "complex perianal fistula*") OR "Perianal disease") OR "fistulizing disease") OR "Fistula-in-ano")) AND cryptoglandular)) AND (((((((((("Rectal Fistula"[Mesh]) OR "perianal fistula") OR "perianal fistula*") OR "complex anal fistula*") OR "complex perianal fistula*") OR "Perianal disease") OR "fistulizing disease") OR "Fistula-in-ano")) AND "Cryptoglandular")) NOT ((((("Inflammatory Bowel Diseases"[Mesh]) OR "Inflammatory Bowel Disease*") OR "IBD")) OR ((("Crohn Disease"[Mesh]) OR "Crohn Disease") OR "Crohn's Disease")) |
| #7 | #3 OR #6 | ((((((((((("Rectal Fistula"[Mesh]) OR "perianal fistula") OR "perianal fistula*") OR "complex anal fistula*") OR "complex perianal fistula*") OR "Perianal disease") OR "fistulizing disease") OR "Fistula-in-ano")) AND ((("Crohn Disease"[Mesh]) OR "Crohn Disease") OR "Crohn's Disease"))) OR ((((((((((((("Rectal Fistula"[Mesh]) OR "perianal fistula") OR "perianal fistula*") OR "complex anal fistula*") OR "complex perianal fistula*") OR "Perianal disease") OR "fistulizing disease") OR "Fistula-in-ano")) AND cryptoglandular)) AND (((((((((("Rectal Fistula"[Mesh]) OR "perianal fistula") OR "perianal fistula*") OR "complex anal fistula*") OR "complex perianal fistula*") OR "Perianal disease") OR "fistulizing disease") OR "Fistula-in-ano")) AND "Cryptoglandular")) NOT ((((("Inflammatory Bowel Diseases"[Mesh]) OR "Inflammatory Bowel Disease*") OR "IBD")) OR ((("Crohn Disease"[Mesh]) OR "Crohn Disease") OR "Crohn's Disease"))) |
| #8 | Surgcial intervention, surgical procedures | (((((("Surgical Procedures, Operative"[Mesh]) OR "Elective Surgical Procedures"[Mesh]) OR "surgical intervention") OR "surgical") OR "surgeries") OR "surgery") OR surger* |
| #9 | Mesemchymal stem cell, stem cell therapy, darvadstrocel | ((("Mesenchymal Stem Cells"[Mesh]) OR "Autologous adipose-derived stem cells") OR "Stem Cell Therapy") OR "darvadstrocel" |
| #10 | #8 OR #9 | (((((((("Surgical Procedures, Operative"[Mesh]) OR "Elective Surgical Procedures"[Mesh]) OR "surgical intervention") OR "surgical") OR "surgeries") OR "surgery") OR surger*)) OR (((("Mesenchymal Stem Cells"[Mesh]) OR "Autologous adipose-derived stem cells") OR "Stem Cell Therapy") OR "darvadstrocel") |
| #11 |  | #7 AND #10  ((((((((((((("Rectal Fistula"[Mesh]) OR "perianal fistula") OR "perianal fistula*") OR "complex anal fistula*") OR "complex perianal fistula*") OR "Perianal disease") OR "fistulizing disease") OR "Fistula-in-ano")) AND ((("Crohn Disease"[Mesh]) OR "Crohn Disease") OR "Crohn's Disease"))) OR ((((((((((((("Rectal Fistula"[Mesh]) OR "perianal fistula") OR "perianal fistula*") OR "complex anal fistula*") OR "complex perianal fistula*") OR "Perianal disease") OR "fistulizing disease") OR "Fistula-in-ano")) AND cryptoglandular)) AND (((((((((("Rectal Fistula"[Mesh]) OR "perianal fistula") OR "perianal fistula*") OR "complex anal fistula*") OR "complex perianal fistula*") OR "Perianal disease") OR "fistulizing disease") OR "Fistula-in-ano")) AND "Cryptoglandular")) NOT ((((("Inflammatory Bowel Diseases"[Mesh]) OR "Inflammatory Bowel Disease*") OR "IBD")) OR ((("Crohn Disease"[Mesh]) OR "Crohn Disease") OR "Crohn's Disease"))))) AND ((((((((("Surgical Procedures, Operative"[Mesh]) OR "Elective Surgical Procedures"[Mesh]) OR "surgical intervention") OR "surgical") OR "surgeries") OR "surgery") OR surger*)) OR (((("Mesenchymal Stem Cells"[Mesh]) OR "Autologous adipose-derived stem cells") OR "Stem Cell Therapy") OR "darvadstrocel")) |

Limits: Clinical Study, Clinical Trial, Clinical Trial Protocol, Clinical Trial, Phase I, Clinical Trial, Phase II, Clinical Trial, Phase III, Clinical Trial, Phase IV, Comparative Study, Congress, Controlled Clinical Trial, Corrected and Republished Article, Dataset, Electronic Supplementary Materials, English Abstract, Evaluation Study, Government Document, Guideline, Journal Article, Observational Study, Randomized Controlled Trial, Technical Report,, published in the last 10 years, Humans, English, Adult: 19+ years, Young Adult: 19-24 years, Adult: 19-44 years, Middle Aged + Aged: 45+ years, Middle Aged: 45-64 years, Aged: 65+ years, 80 and over: 80+ years
